# Supplementary material for: An inexpensive, customizable microscopy system for the automated quantification and characterization of multiple adherent cell types
Source: PeerJ. 2018 Jun 5;6:e4937. doi: 10.7717/peerj.4937 (PMC5993021; doi:10.7717/peerj.4937)
Supplement: Supplemental Information 1 — Recommendations for optimizing workflow, handling of multinucleated cells, processing of tissue samples, and analysis of cells in suspension are discussed here. An explanation of CellProfiler codes and associated functions can also be found here. [file peerj-06-4937-s001.docx]

**Supplementary**

**An Inexpensive, Customizable Microscopy System for the Automated Quantification and Characterization of Multiple Adherent Cell Types**

Vishwaratn Asthana^1*^, Yuqi Tang^1¶^, Adam Ferguson^1¶^, Pallavi Bugga^1^, Anantratn Asthana^2^, Emily R. Evans^1^, Allen L. Chen^1^, Brett S. Stern^1^, Rebekah A. Drezek^1^

**^1^** Department of Bioengineering, Rice University, Houston, Texas, United States of America,

**^2^** Berkeley University, Berkeley, California, United States of America

^*^ Corresponding author

E-mail: [vishwaratn.asthana@rice.edu](mailto:Vishwaratn.asthana@rice.edu) (VA)

^¶^ These authors contributed equally to this work.

**Suggestions for Optimized Workflow**

After performing several whole well imaging runs, we have several recommendations for obtaining optimal results. First, we suggest imaging cells at a magnification of 10x or less in order to speed up image capture times and keep the cells as evenly illuminated as possible. Second, plate bottoms should be wiped with ethanol before each imaging run to remove debris and prevent light artifacts from skewing subsequent analysis. Third, while images can be stitched together during image acquisition, we suggest for the analysis stage that whole well images be cropped into smaller subset images that are no larger than 5,000 by 5,000 pixels, but no less than 500 by 500 pixels. We have found that when the images are too large, the process of determining an accurate threshold for each image in order to discriminate foreground from background becomes inefficiently long. By breaking up the image, local thresholding is quicker and image analysis can be parallelized and allocated to multiple processers. Images should not be too small however, as cells that are at the boundary are more likely to be doubly counted if CellProfiler is set to retain cells at the image border, or more likely to be tossed out if cells at the image border are set to be excluded.

Another item to note is that with whole-well fluorescent imaging, conventional polystyrene multiwell plates often have a ring of fluorescence around the well edge due to the autofluorescent properties of the plastic. Simply cropping out the fluorescent ring is not sufficient because most cropping algorithms will create a series of zeros outside of the crop zone. This creates a large gradient in intensity at the crop boundary, which can cause CellProfiler and most cell segmentation softwares that detect changes in intensity gradient to incorrectly treat the crop zone as a series of cells. To overcome this, the Rolling Ball algorithm available on ImageJ can be used to correct the unevenly illuminated background by taking a local average background value around every pixel and subtracting it from the original image (Sternberg 1983). A second option would be to use black-coated multiwell plates to prevent autofluorescence of the plastic, however, this was not tested in the present study.

Lastly, we present an alternative algorithmic approach users may consider for quantifying multiple adherent cells that preserves the power of nuclear segmentation but is less computationally demanding. The method involves overlaying the mask of each segmented nucleus onto to the corresponding surface or cytoplasmic stained image. If the surface or cytoplasmic fluorescent stain intensity within the region demarcated by the segmented nucleus exceeds a certain threshold, then the nucleus can be assigned to the cell population stained with that particular dye. These nuclei can subsequently be counted in a manner similar to flow cytometry. Overall, this approach is less computationally intensive and does not necessarily require homogenous whole-cell staining. However, as can be seen in Figure 6, the region of the cell where the nucleus resides can sometimes stain poorly, which can cause the fluorescent intensity in the region demarcated by the nucleus to fall below the necessary threshold, and subsequently result in these cells being missed. Using the current approach with inclusive whole-cell masks, holes in the cell can be filled, thus circumventing this problem. Determination of which option should be chosen can be made based on the user’s needs.

**Performance Relative to Alternative Cell Counting Systems**

The proposed microscopy-based cytometer is not the only option for quantifying individual cells including in a co-culture system. As stated earlier, flow cytometry is a powerful technique for quantifying multiple cell populations in an experimental setup. However, depending on the requirements of the experiment and resources available in the lab, flow cytometry may not always be ideal. Because flow cytometry requires cells to be brought into suspension for analysis, cells or extracellular markers that would be damaged by trypsin as well as strongly adherent cells that would require cell scraping can not reliably be measured. In addition, certain types of data, such as spatial distribution and cell migration, are lost when cells are brought into suspension. Flow cytometry also obtains relative counts as opposed to absolute counts, making the quantification of multiple shifting cell populations in an experiment difficult to assess without the use of an external normalizing control. To obtain absolute counts using single-platform flow cytometry, one can perform volumetric analysis; however, this requires a completely uniform cell suspension that is free of air bubbles. This type of suspension can be difficult to obtain (Pappas & ebrary Inc. 2010; Sklar 2005). An alternative, more reliable option is to spike the solution to be analyzed with counting beads at a known concentration. The beads serve as a reference to tabulate the absolute count of cells passing through the instrument. Counting beads, however, can introduce experimental error via pipetting, insufficient mixing, and bead settling (Brando et al. 2000). Their use also requires an additional step in sample processing. We demonstrate that the microscopy-based cytometer is capable of quantifying cells without bringing them into suspension. In addition to the linear curves generated in Figure 3, a comparative test of the microscopy-based cytometer to a conventional hemocytometer reveals nearly identical quantitative performance indicating the system is also capable of obtaining absolute cell counts, even for those cells that lie near the well edge (Fig S2).

Many commercial optical cytometers are also available for cell quantification, such as the Cytation 5, Celligo Image Cytometer, Tali Image Cytometer, and Cell Cytel Imaging System, as well as laser scanning cytometers. While these platforms address many of the limitations of flow cytometry, they are still hindered in their overall sensitivity, dynamic range, and applicability in co-culture setups. While some commercial cytometers allow for direct quantification of adherent cells on multi-well plates, many systems, such as the Tali Image Cytometer for instance, require cells to be in suspension prior to quantification, and thus necessitate trypsinization or manual scraping for adherent cells.

In regards to the dynamic range afforded by each system, most commercial cytometers (including the Celligo, Cytation 5, Cell Cytel, and Tali cytomers) have a working range somewhere between 2 to 4 orders of magnitude. The Cytation 5, for instance, reports a dynamic range between 200 to 25,000 cells/mL for 96- well plates, with cell counts plateauing above 25,000 cells/mL. In addition, many current commercial optical cytometers appear to struggle with quantification near the cell confluency limit. This is unlike the optical system presented here, in which cell counts scaling a 5-log dynamic range can be quantified with remarkably high accuracy, even near the confluency limit of the well. The single-cell sensitivity of our microscopy-based cytometer is also a significant improvement over the reported sensitivities of many commercially available cell cytometric systems.

One of the most advantageous aspects of the microscopy-based cytometer presented here is its ability to accurately quantify multiple adherent cell types in a co-culture setup. As stated in the discussion section of the paper, the microscopy-based cytometer can reliably quantify multiple cells of various morphologies using a unique combination of cell fluorescent staining and image processing steps in order to generate cell-specific masks that assign nuclei seeds to their respective cell populations. This method generates minimal error when compared to the primary segmentation method that most commercial cytometers rely on.

While co-culture studies are increasingly being recognized as essential tools for recreating *in vivo* conditions, very few labs are conducting them; this holds especially true for co-culture studies in which cells are in direct physical contact. Current literature suggests that many labs are still either using transwell inserts or microfluidic chambers to physically separate cells, or instead exchanging conditioned media between two independent cell flasks (Arrigoni et al. 2016; Goers et al. 2014; Katt et al. 2016). Though theoretically capable of quantifying multiple adherent cell types, commercial optical counting systems are not being used for co-culture, perhaps due in part to the limitations described above. The steep overhead costs of such systems may also act as a barrier. Most commercial optical counters cost anywhere upwards of $150,000. For labs that already possess a basic fluorescent microscope however, the proposed microscopy-based cytometer requires only the addition of a motorized x-y-z stage (for automated whole-well scanning) and z-axis piezoelectric (for automated focus). This amounts to a roughly $20,000 investment (component parts from Thorlabs) for a system that can also function as a flow cytometer.

A review of recent literature that cites the use of commercially available optical counters reveals that these systems are still primarily used for quantifying mono-cultures, but not co-cultures. Accordingly, there appears to be a significant need for cytometric systems (such as the one presented here) that are capable of accurately quantifying multiple adherent cell types in co-culture setups.

**Microscopy-Based Cytometer** **Technical Specifications**

The microscopy-based cytometer system discussed in this paper has relatively minor requirements. A fluorescent microscope with motorized x, y, and z stage and associated microscopy software to manipulate the motors is a prerequisite for large-scale, automated imaging. Sufficient storage capacity to save the images and a power PC for image analysis is also recommended. On average, microscope acquisition times for a standard multiwell plate run anywhere from 30 minutes to 3 hours depending on how many different fluorescent channels need to be used and the exposure time. Cell fixation prior to imaging helps preserve cellular morphology and prevent cell detachment during extended imaging runs. Subsequent image processing via ImageJ and segmentation using CellProfiler are typically left overnight, but single fluorescent channel, whole-well plate images, which are usually 20-30 gigabytes in size depending on image resolution, take approximately 1-2 hours to analyze on a high-end computing platform. While the entire process is relatively long compared to most traditional cell viability assays, the method is entirely automated and requires no user oversight once running.

**Explanation of CellProfiler Codes and Associated Functions**

For nuclei identification alone, the following functions were performed in CellProfiler: 1) illumination correction using *CorrectIlluminationCalculate* and *CorrectIlluminationApply*, 2) primary object identification using *IdentifyPrimaryObjects*, and 3) counts export and mask saving using *ExportToSpreadsheet* and *SaveImages* respectively (S1 CellProfiler Code).

For surface and cytoplasm stains, Ilastik, a software that comes prepackaged with CellProfiler, was used first to train and develop a classifier to accurately identify regions of cellular fluorescence. A classifier for relevant cell type and stain combinations were made and imported into CellProfiler. The following functions were then performed in CellProfiler: 1) illumination correction using *CorrectIlluminationCalculate* and *CorrectIlluminationApply*, 2) cell classification of the cytoplasmic and/or surface stain using *ClassifyPixels*, 3) primary object identification to fill in any holes from the cell classification function using *IdentifyPrimaryObjects*, 4) generating an inclusive mask and overlaying it on the nuclear stain using *MaskImage*, 5) primary object identification of nuclei present within the masked region, 6) secondary object identification to delineate the borders of the surface or cytoplasmic stain using *IdentifySecondaryObjects*, 7) removal of overly large cell artifacts occasionally generated via *IdentifySecondaryObjects*, that can interfere with functions that obtain cell size or fluorescent intensity but not counts, using *MeasureObjectSizeShape* then *FilterObjects*, 8) calculating the fluorescent intensity, object area, and nearest neighbor of secondarily defined objects using *MeasureObjectIntensity*, *MeasureObjectSizeShape*, and *MeasureObjectNeighbors* respectively, 9) and counts export and mask saving using *ExportToSpreadsheet* and *SaveImages* respectively (S2 CellProfiler Code). The algorithmic workflow for multi-stain co-culture studies is visually depicted in Figure S3.

It should be noted that the provided code has been optimized for CellProfiler version 2.2.0 and may need modifications or not be compatible with earlier or later versions of CellProfiler. Previous releases of CellProfiler can be found at http://cellprofiler.org/previous_releases/.

**Options for Multiplexing**

One of the strongest attributes of the microscopy-based cytometer is its ability to quantify multiple cell types. In addition to the Vybrant CFDA SE dye, which is green, several alternative vital dyes can be used to stain the cell cytoplasm and increase the number of unique cell types being assayed. These include other dyes in the succinimidyl ester series such as Marina Blue, which is blue, and SNARF-1 carboxylic acid, which is red. Like Vybrant CFDA SE, these dyes are activated by esterases expressed in the cell cytoplasm, and can be used to increase the number of differentially labeled cells in a co-culture setup. Alternatively, dyes of the CellTracker series (Thermo Fisher Scientific), the majority of which are activated by glutathione S-transferase, can also be used, and include dyes that fluoresce in the blue, violet, green, orange, red, and deep red spectrum. These long-lived dyes are also multigenerational, well-retained, and nontoxic, making them ideal for extended co-culture studies (Gross et al. 1995). High Content Screening (HCS) CellMask and NuclearMask (Thermo Fisher Scientific) stains can also be used to label the cytoplasm and nucleus respectively with various colors. However these dyes are not vital and are usually added after cell fixation, implying that they cannot be used to differentially label cells. The DiI dye series is also an option for long-term cell staining, however we have had more success using esterase-activated cytoplasmic stains and not lipophilic tracers, because the staining is generally more homogenous.

Although not explored in this study, viral transduction is another common, though initially tedious method, for long-term staining of cells. Viral transduction can be used to stain either the nucleus and/or cytoplasm permanently with various fluorescent reporters, while still maintaining cell viability. Viral transduction is especially powerful because it is the only option for long-term staining of the nucleus. Nuclear staining using dyes like Hoechst can be performed on live cells; however, stained cells need to be analyzed relatively quickly since most classes of nuclear dyes intercalate with the genome, detrimentally affecting transcription and replication (Trotta et al. 2003). Expression of fluorescent reporter proteins that localize to the nucleus or cytoplasm using viral transduction preserves cell viability and makes previously unfeasible, extended co-culture time course studies a possibility.

Using various combinations of dyes it is possible to increase the number of parameters being assessed in a single experimental setup, or the number of cells being co-cultured and quantified. Generally the number of parameters/cells that can be assessed is **n-1** the number of available dyes and corresponding fluorescent channels. It should theoretically be possible however to increase the number of unique cell populations being quantified by using a barcode approach to labeling cells. For example, two unique cytoplasmic stains such as Vybrant CFDA SE and SNARF-1 carboxylic acid can be used to label three cells instead of just two by labeling an additional cell population with both Vybrant and SNARF-1 carboxylic acid. During image processing, only nuclei that lay within the boundary mask delineated by both the green and red channel would be considered when quantifying the third cell population.

The same approach could be used with antibody staining as well. Primary antibody labeling using readily available dyes has become a relatively straightforward process using fast conjugation kits. Antibodies targeting a single antigen can be split into two or more groups and each be labeled with a unique fluorophore. The targeted cell type expressing the unique antigen would illuminate in multiple channels and only nuclei residing within the multicolored cells would be considered. Overall, such a barcoding approach allows for **2^(n-1)^-1** unique labels where again **n** is the number of available dyes and corresponding fluorescent channels. It should be noted that one fluorescent channel must be dedicated to imaging the nuclei so it cannot be used for combinatorially labeling cells, something that is taken into account in the formula above.

A barcoding approach to cell quantification was not tested directly in this study, but in the triply stained J774.A1 cells, there was only a marginal loss in accuracy when counting nuclei that lay within the boundary mask delineated by both the Vybrant and anti-CD11b antibody stains with performance primarily limited by the least accurate stain (Fig S4). It should be noted that a deterioration in performance may be observed when the number of stains on a single cell type increases, especially if the stain types overlap. Cells may experience increased toxicity when labeled with an increasing number of vital cytoplasmic dyes, and a potential loss in fluorescent signal over background when splitting up antibody labels. Testing a range of stain concentrations and incubation times for an optimum should, however, address these issues. New exciting options for multiplexing are also in development including fluorescent lifetime imaging and quantum dots, which will dramatically increase the total number of parameters/cells that can be assessed in a single experiment (Arya et al. 2005; Bastiaens & Squire 1999; Becker 2012; Ding et al. 2015; Lee-Montiel et al. 2015; Michalet et al. 2005; Pinaud et al. 2006).

**Handling of Multinucleated Cells**

Though not an issue with the cell types that were utilized in this paper, multinuclear cells pose a challenge for the microscopy-based cytometer. The system depends strongly on the stoichiometric relationship between nuclei and cell count to accurately quantify cells. The presence of multinuclear cells or the induction of nuclear fragmentation can cause cellular counts to be overrepresented to varying degrees. The presence of multiple nuclei is seen naturally in certain cells, such as osteoclasts and skeletal myocytes, and unnaturally as well as sporadically in cancer (Krajcovic et al. 2011; Weihua et al. 2011). Multinucleation and/or nuclear fragmentation can also be induced using pharmacological compounds that inhibit cell division, such as chemotherapeutics (An et al. 2010; Wen et al. 2016).

If the presence of multiple nuclei is suspected, additional algorithmic steps can be taken to ensure that the correct number of cells is counted. First, cells must be stained with both DAPI and a cell body stain. Cell segmentation of whole cells generally fail due to complex morphologies or cell-cell contact, however primary object identification of whole cells can be used to identify cell objects that contain more than one nucleus, and remove these extra nuclei from subsequent analysis. This can be done by pairing the independently identified cell objects with the nuclear objects contained within them. If more than one nucleus is paired with a single cell object, then all but one of the paired nuclei can be removed, after which CellProfiler will return to normal analysis using nuclei as seeds. While primary object identification of whole cells is not ideal, it performs well enough to identify the majority of the cell body, making it ideal for removing extraneous nuclei that could interfere with analysis. Alternatively, if the percent of multinucleated cells or extent of multinucleation is of interest, a similar approach can be used to determine these parameters as well.

**Processing of Tissue Samples**

In addition to *in vitro* co-culture experiments, the microscopy-based cytometer should also be capable of analyzing complex tissue samples. Using the same general procedure of multiplex staining, mask generation, and nuclear segmentation, many cell types can be enumerated with high precision and statistical confidence over a large region of tissue. Parameters like DNA, RNA, or protein expression, target co-expression and co-localization, and cellular morphology as well as spatial distribution can also be extracted, which may prove especially useful when studying, for example, tumor heterogeneity or tumor immune cell infiltration. Adjunct protocols such as laser capture microdissection are also an option, and can be paired with additional forms of analysis such as Next Generation Sequencing (Espina et al. 2006). Multiplex staining of a single tissue specimen has the added benefit of preserving precious clinical sample.

Analysis of tissue specimens differs slightly from *in vitro* cell culture analysis. Samples will generally require more preprocessing, staining using antibodies instead of vital dyes, and a relatively thin and flat section for in focus, automated, large-scale acquisition. The manner in which samples are processed is also important and can affect staining quality and subsequent imaging. Though imaging of tissue samples was not tested in the present study, discrepancies in image quality and character from standard cell culture imaging can be accounted for by adjusting the established custom segmentation algorithms.

**Recommendations for Analyzing Poorly Adherent Cells and Cells in Suspension**

Poorly adherent cells that would likely detach during processing can be anchored down strongly using a fibronectin or poly-L-lysine coated plate (Cooke et al. 2008; Mazia et al. 1975). For cells in suspension, we recommend processing the cells in solution first, much like in flow cytometry, then anchoring them down for imaging. This can be done by centrifuging cells onto a poly-L-lysine coated surface using a centrifuge adaptor for multiwell plates or Cytospin for slides (Arora et al. 2011; Koh 2013). Alternatively, cells in suspension can be placed under a coverslip for analysis.
