# Supplementary figures and images for: An inexpensive, customizable microscopy system for the automated quantification and characterization of multiple adherent cell types
Source: PeerJ. 2018 Jun 5;6:e4937. doi: 10.7717/peerj.4937 (PMC5993021; doi:10.7717/peerj.4937)

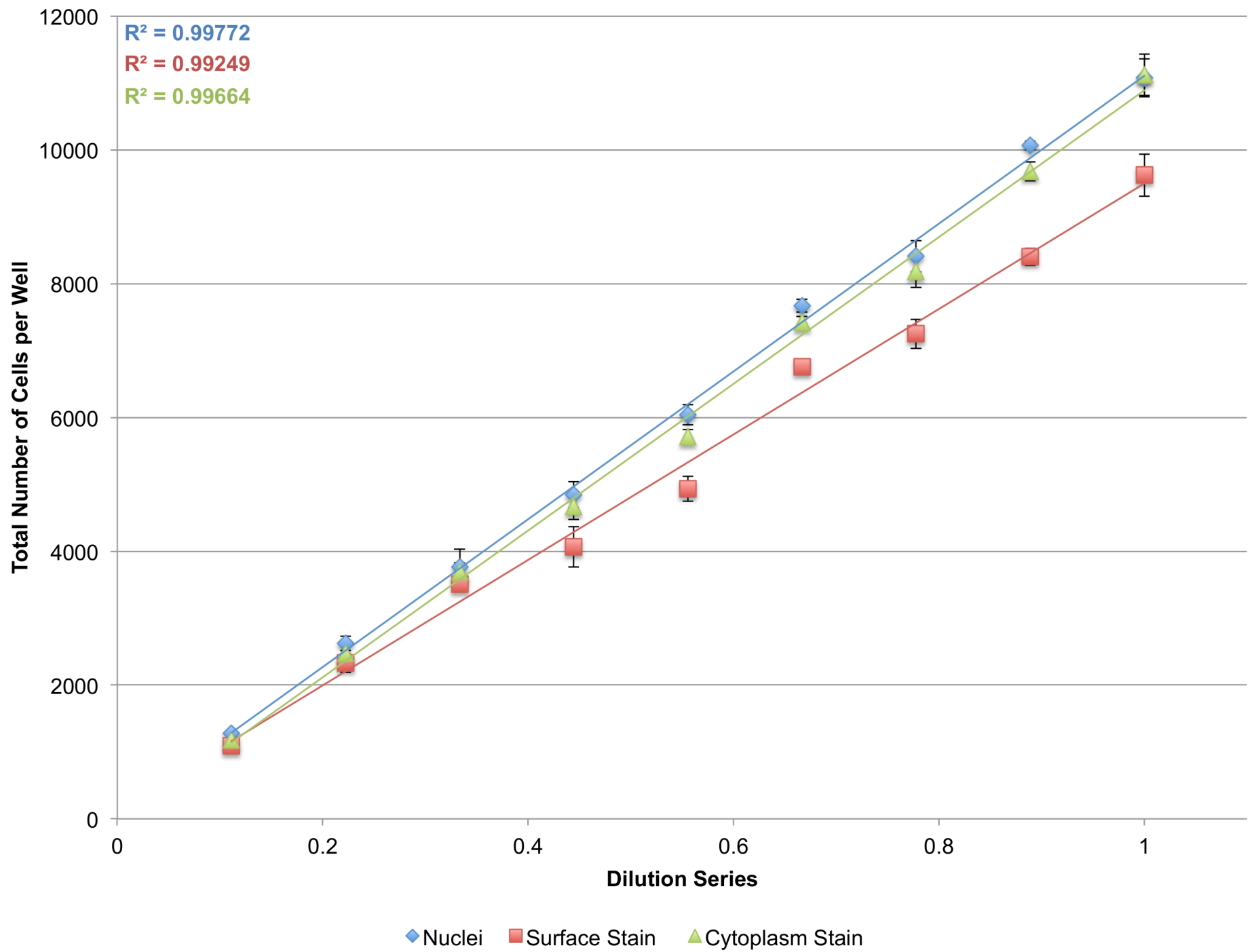

Supplement: Supplemental Information 5 — The same linear dilution series of J774.A1 cells that was used to assess the accuracy of secondary counts generated using nuclei as seeds in Figure 5 was instead primarily segmented. Cells were plated starting from 10,000 cells/well down to 1,000 cells/well on a 48-well plate and were stained with Vybrant CFDA SE (cytoplasmic stain), phycoerythrin (PE)-conjugated anti-CD11b antibodies (surface stain), and DAPI (nuclear stain). The system performs less than ideally when the fluorescent outline of the cell is used to identify cells instead of their nuclei and would likely have performed significantly worse had the J774.A1 cells not been relatively round. Error bars represent the standard deviation between triplicate conditions. [file peerj-06-4937-s005.pdf]

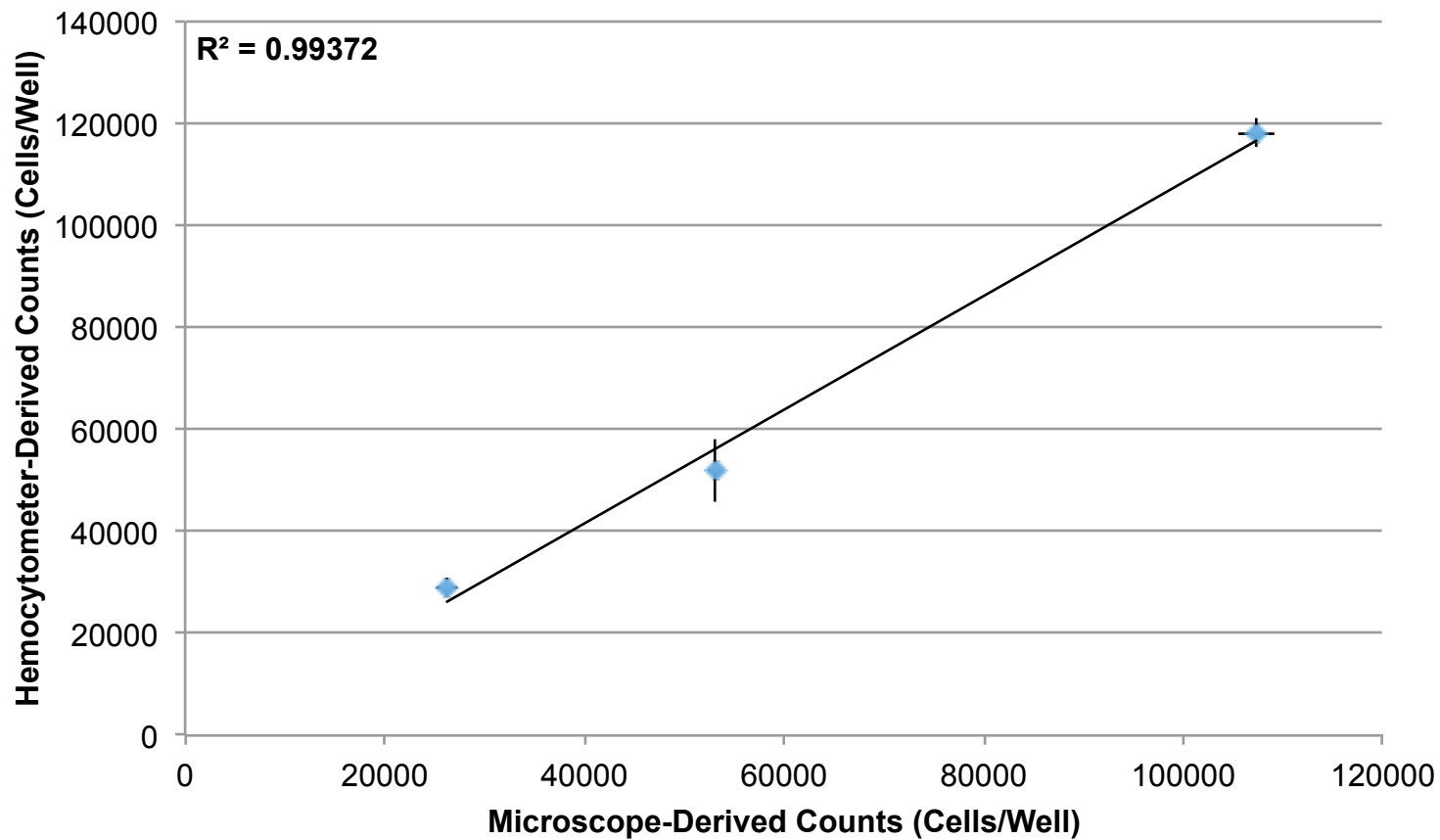

Supplement: Supplemental Information 6 — A dilution series of WPMY-1 cells going from 100,000 cells/well down to 25,000 cells/well was seeded into two 24-well plates and given 24 hours to adhere to the surface. Cell nuclei from the first plate were stained with DAPI then quantified using the microscopy-based cytometer, while cells from the second plate were brought into suspension using trypsin and quantified using a hemocytometer. The two systems perform comparably and obtain similar cell counts (R2 = 0.99, slope = 1.12) validating the ability of the microscopy-based cytometer to obtain absolute cell counts. Error bars represent the standard deviation between triplicate conditions. [file peerj-06-4937-s006.pdf]

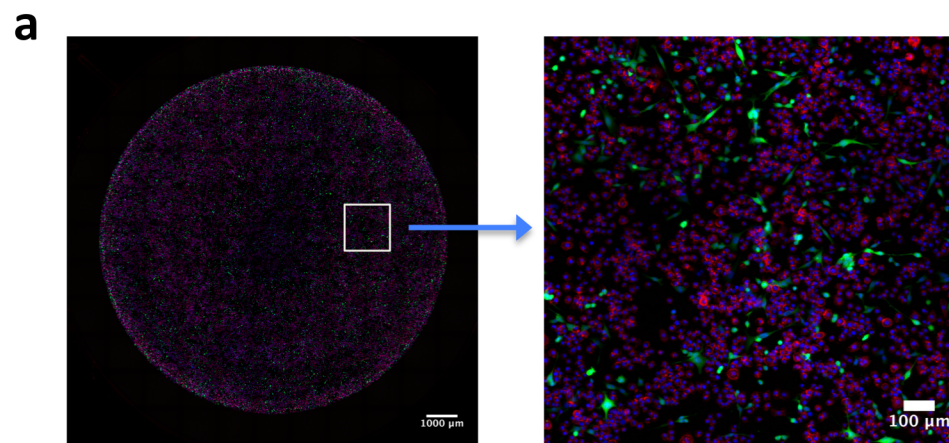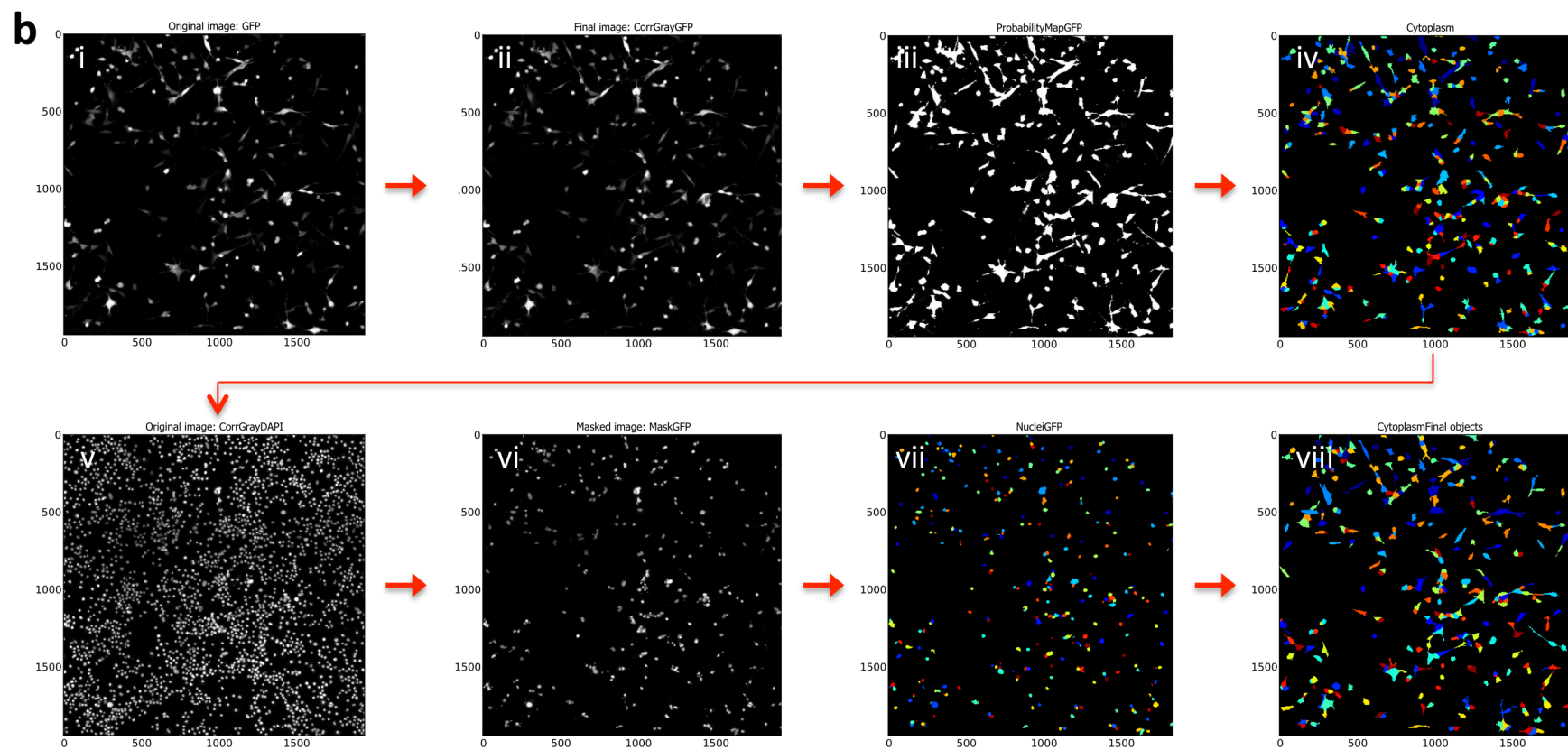

Supplement: Supplemental Information 7 — (a) J774.A1 macrophages labeled with PE-conjugated anti-CD11b antibodies (red surface stain) were co-cultured with JC CRL 2116 tumor cells labeled with Vybrant (green cytoplasmic stain). Both cells were also stained with DAPI. (b) The representative image shown in (a) was then run through CellProfiler for processing. For demonstrative purposes, only the Vybrant stained JC CRL 2116 cells are shown in the sample workflow. First, illumination correction is performed on the i) original grayscale image to ii) correct for non-uniformities in illumination. iii) Cell classification of the cytoplasmic stain is then used to identify areas of fluorescence that correspond to the cell body. iv) Primary object identification is then used to fill in any holes generated during cell classification. The subsequently generated image serves as an inclusive mask that is applied to the v) original DAPI image in order to produce a new image vi) that contains only nuclei belonging to Vybrant stained cells. vii) Primary object identification is used once again to identify and quantify the remaining nuclei which then act as seeds for secondary object identification and cell body delineation. [file peerj-06-4937-s007.pdf]

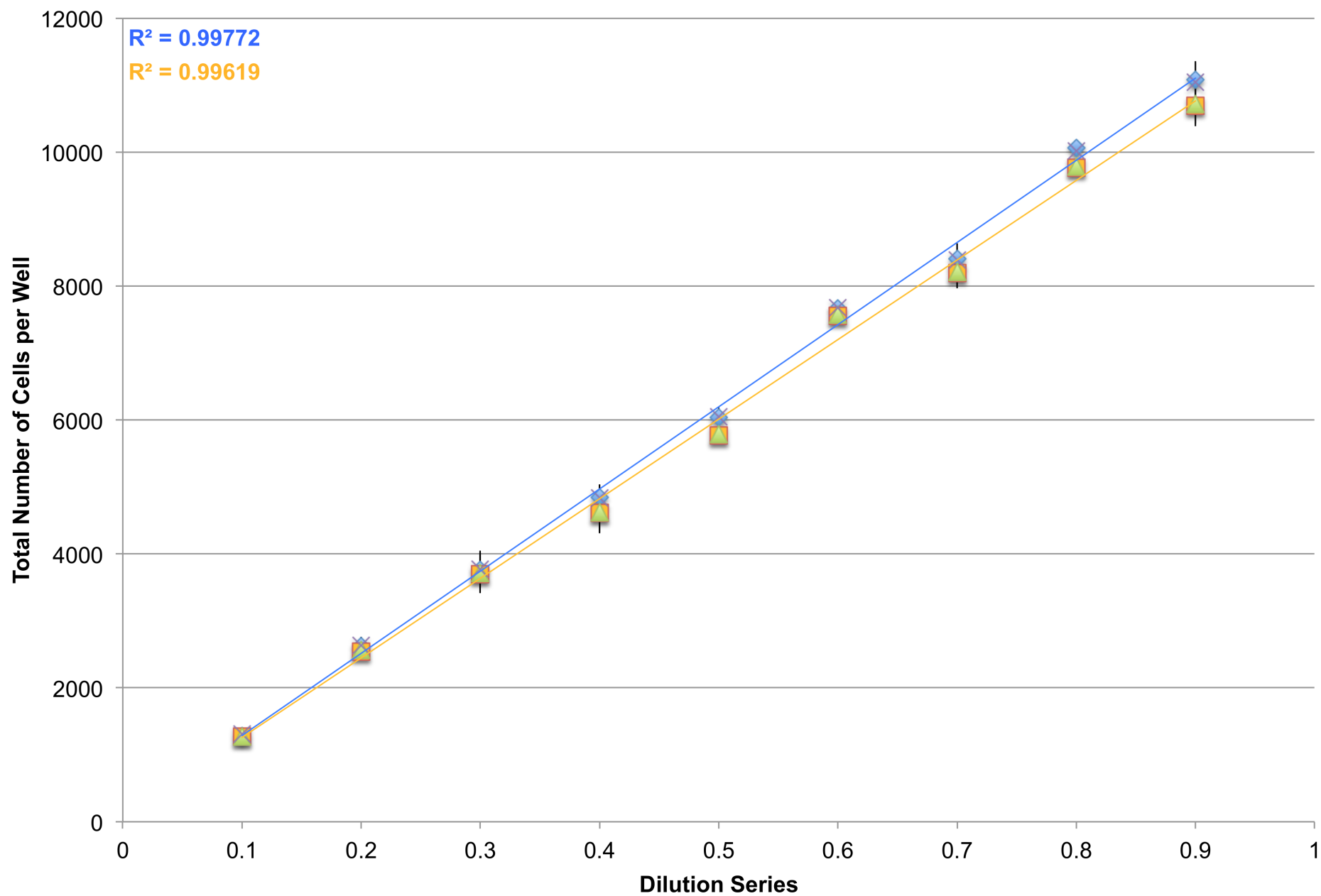

◆ Nuclei Alone      □ Double Mask      ▲ Antibody Mask  
× Cytoplasm Mask      — Linear (Nuclei Alone)      — Linear (Double Mask)

Supplement: Supplemental Information 8 — The same linear dilution series of J774.A1 cells that was used to assess the accuracy of secondary counts generated using nuclei as seeds in Figure 5 was instead used to assess the performance of a barcode approach to multiplex cell quantification. Cells were plated starting from 10,000 cells/well down to 1,000 cells/well on a 48-well plate and were stained with Vybrant CFDA SE (cytoplasmic stain), phycoerythrin (PE)-conjugated anti-CD11b antibodies (surface stain), and DAPI (nuclear stain). Plots of nuclei alone, nuclei delineated by an antibody surface mask, nuclei delineated by a cytoplasm mask, and nuclei delineated by both an antibody surface as well as cytoplasm mask were generated. There is only a marginal loss in performance when counting nuclei demarcated by two masks with accuracy primarily limited by the least accurate stain. Error bars represent the standard deviation between triplicate conditions. [file peerj-06-4937-s008.pdf]
